# All-weather precision alignment technology for ultra-wide steel box girders in cable-stayed bridges

**DOI:** 10.1038/s41598-025-08562-6

**Published:** 2025-07-11

**Authors:** Wang Laifa, Li Yexun, Zou Ci, Zhou Sicheng

**Affiliations:** CCCC Third Harbor Engineering Co., Ltd, Shanghai, 200030 China

**Keywords:** Cable-stayed bridge, Precision alignment, Dynamic, Steel box girder, Elevation, Civil engineering, Mathematics and computing

## Abstract

High precision is required during the precise matching construction of steel box girder cable-stayed bridge segment suspension assembly. Due to temperature differences, sunlight, and other factors, the bridge shape changes dramatically during the day, so the precise matching of steel box girder cable-stayed bridges is generally carried out at night when the temperature is stable. However, nighttime construction restricts the progress of the project and poses certain safety hazards. This article proposes a precise matching construction method for steel beams during the dynamic changes in bridge line shape during the daytime through theoretical calculations and numerical analysis. After one measurement, two retests, and three calculations, and considering the influence of process and environmental factors, dynamic measurement, real-time calculation, and real-time control were applied to Yangmeizhou Bridge to achieve all-weather dynamic precise matching construction of steel box girders, verifying the feasibility of this method.

**Supplementary Information:**

The online version contains supplementary material available at 10.1038/s41598-025-08562-6.

## Introduction

The steel box girder structure, owing to its lightweight and high load-bearing capacity, has been extensively utilized in cable-stayed bridges, suspension bridges, and long-span continuous beam bridges^1,2^. During the cantilever assembly process of steel box girders, precise alignment and positioning of the steel girders are typically conducted according to geometric methods. For cable-stayed bridge structures, the bridge alignment is significantly influenced by environmental conditions such as sunlight and temperature variations^3–8^. To ensure the accuracy of the steel girder alignment, measurements and precise alignment construction are usually carried out during the night when the temperature is relatively stable. For instance, the Zhongshan Bridge of the Shenzhen-Zhongshan Link^9^, the Danjiangkou Reservoir Bridge^10^, and the Xijiang Bridge^11^ all underwent precise alignment construction at night. During the construction of the Sutong Bridge, it was also considered that matching the girder segments two hours after sunset could essentially eliminate the adverse effects of temperature on the local alignment^12,13^.

According to the specification “Technical Specifications for Construction Monitoring and Control of Highway Bridges”, the elevation of the main girder is quite sensitive to changes in the temperature field, and measurements are generally taken during periods when the temperature field is relatively stable^14^. However, this approach affects the construction schedule to some extent and increases construction risks. Moreover, even during nighttime construction, the temperature of the steel girders continues to change^15^, and the elevation of the bridge is constantly fluctuating, leading to errors in positioning when using static data^16,17^. The assessment and prediction of dynamic deflection are crucial for the precise positioning of steel box girders^18^.

**Fig. 1:**
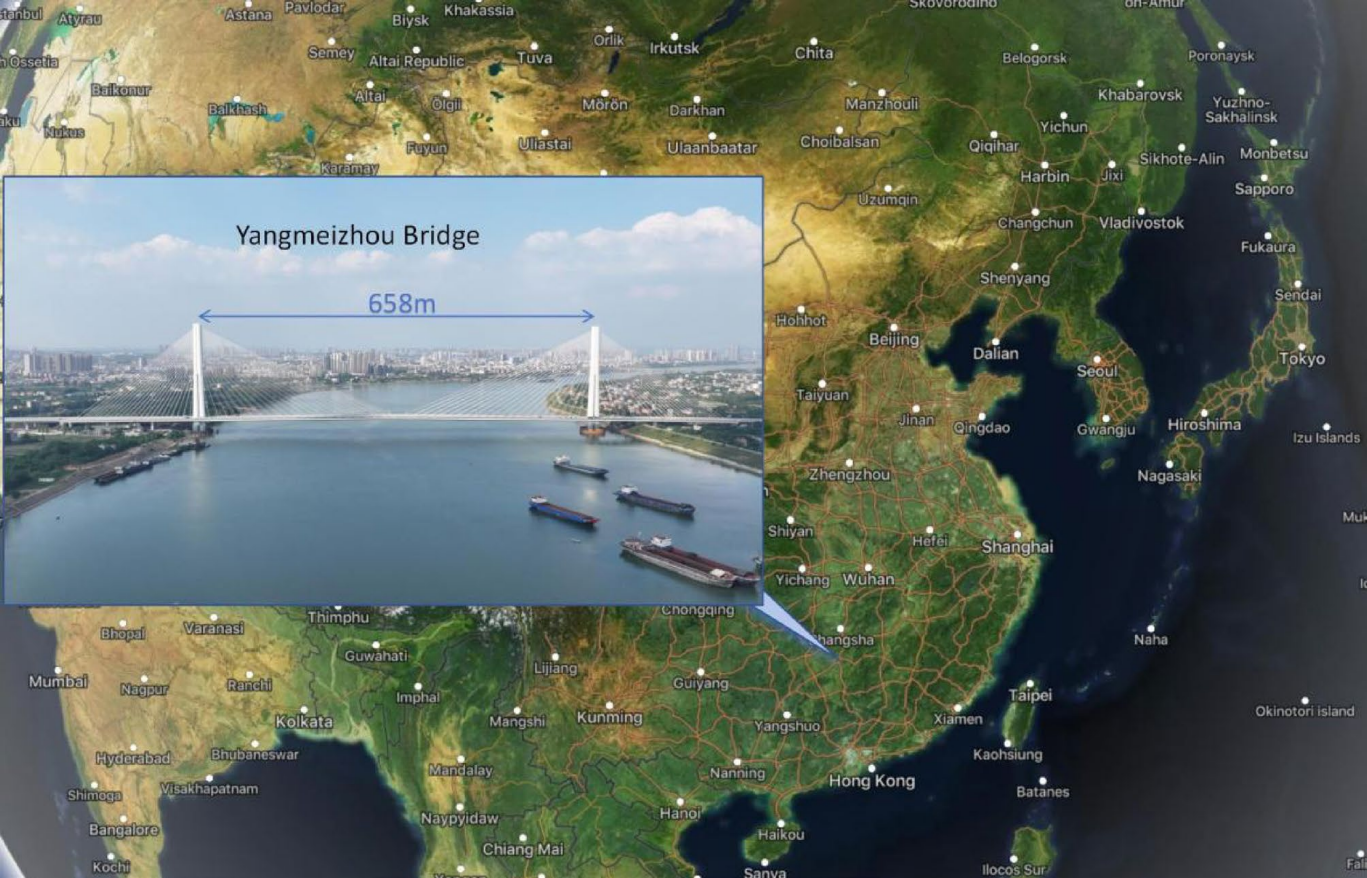
Location Map of Yangmeizhou Bridge. Base map data from MapCarta (https://mapcarta.com), available under the Open Database License (ODbL). Data accessed: May 20, 2025.

**Fig. 2:**
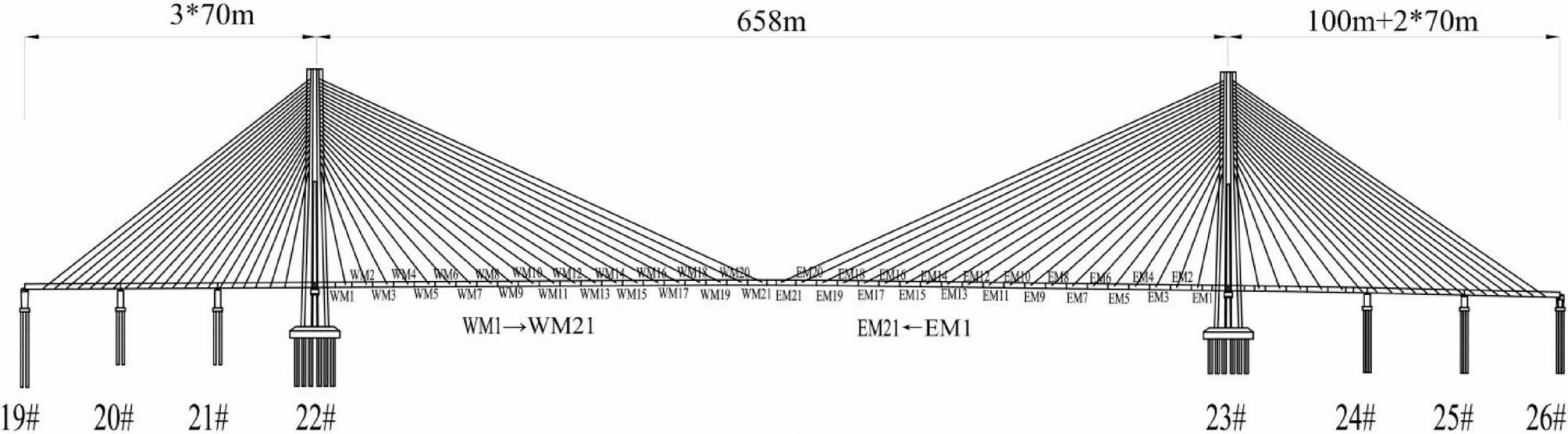
Elevation of Yangmeizhou Bridge.

**Fig. 3:**
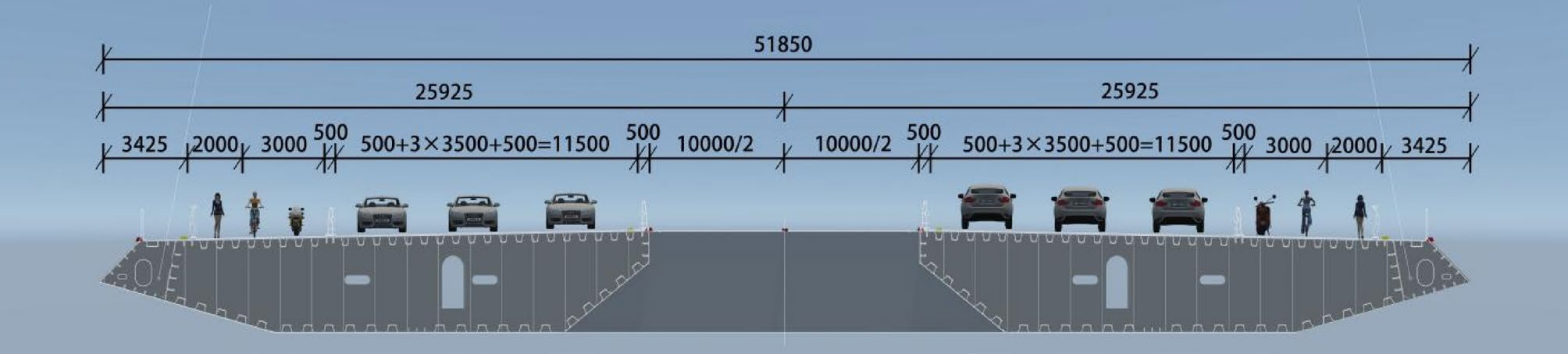
Cross-sectional of Yangmeizhou Bridge.

The Yangmeizhou Bridge is located in Xiangtan, Hunan, China(Fig. 1). It is a double tower double cable plane cable-stayed bridge with a main span of 658 m and a total construction cost of 1.376 billion yuan (Fig. 2). The main girder adopts a steel box girder structure, with standard segment lengths of 15 m, a width of 51.85 m (Fig. 3), and a mass of 340 tons. It employs the cantilever lifting construction method using deck cranes. The total cost of the bridge is 1.376 billion RMB. This paper, through the analysis of steel girder alignment measurement data, proposes a construction control method for the dynamic precise alignment of steel box girders in cable-stayed bridges. This method involves the use of dynamic data for the measurement, positioning, and re-measurement of steel girders, enabling the precise alignment construction of steel box girders to be carried out during the daytime.

## Method

### Dynamic precision alignment construction technique

**Fig. 4:**
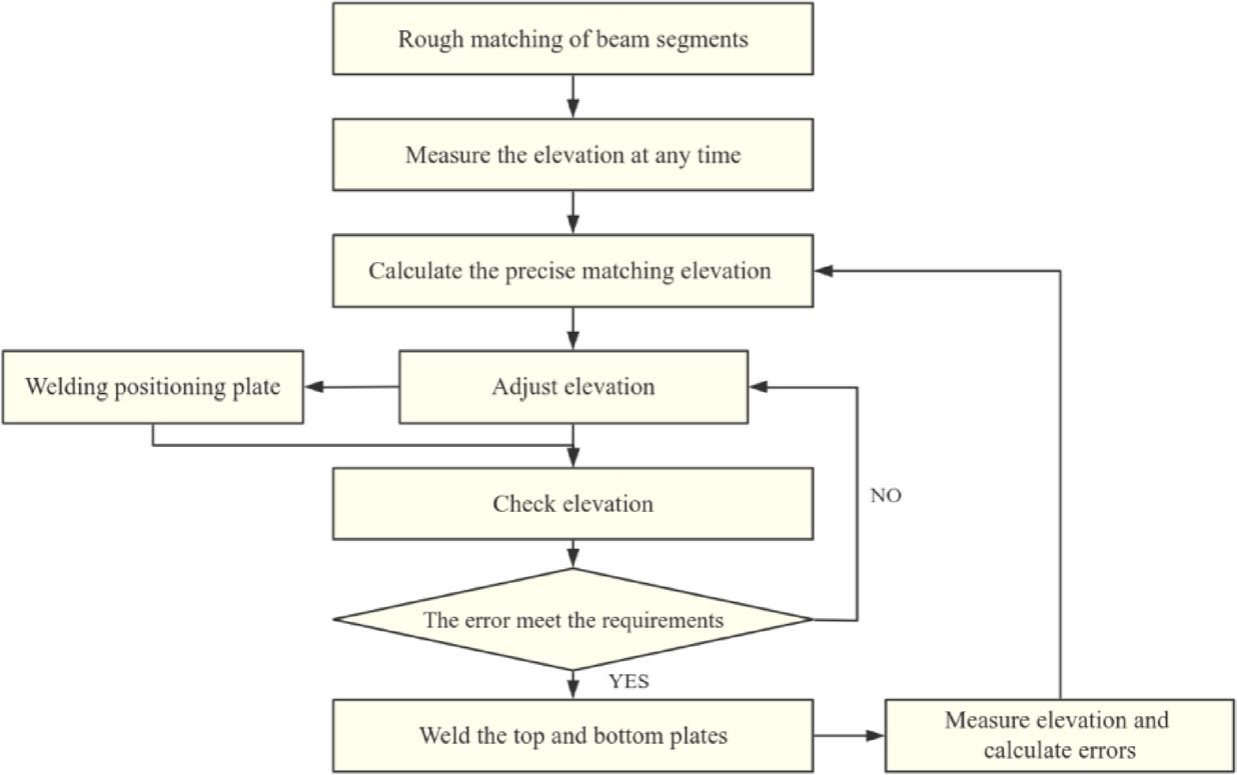
Construction process of dynamic precision matching.

The Yangmeizhou Bridge was constructed using the method described in this article, and its main process is as follows:

The steel beam was lifted into place by the bridge deck; The positioning bolts for the outer web plate position were installed and the steel beam was preliminarily positioned; The elevation of adjacent steel box girders was measured for the first time. Based on geometric relationships and error correction, the precise positioning elevation of the installed steel beams was calculated and adjusted; The positioning steel plate at the belly plate was welded, and the elevation of the adjacent two steel beams and the installed steel beams was measured for the second time. The precise positioning error was calculated and adjusted using the algorithm in this article; If there was an error, a jack was used to adjust the height difference between the top and bottom plates of the steel beams on both sides of the weld to be level, and the top plate of the steel beam was welded; The elevation of the adjacent two steel beams and the installed steel beams was measured for the third time, and the error was calculated according to the algorithm in this article as the construction process error of this section of the beam, providing a basis for error correction for subsequent construction. (Fig. 4)

The steel beam was installed using a bridge deck crane, and the main lifting equipment was two 350 t continuous hydraulic jacks. The elevation of the steel beam was controlled by a hydraulic system with a precision of 1 mm.

### Precise alignment positioning algorithm

Fine matching positioning is actually the process of restoring the manufacturing line shape of steel beams. The manufacturing pre camber can be calculated using finite element software and controlled during steel beam manufacturing^19^.

Measure the installed segments before precise matching, and calculate the accurate position of the segment to be installed based on the relative position of the manufacturing line type.

The process of precise alignment positioning is essentially the restoration of the steel girder’s manufacturing shape. The manufacturing camber can be calculated using finite element software and controlled during the fabrication of the steel girder.

**Fig. 5:**
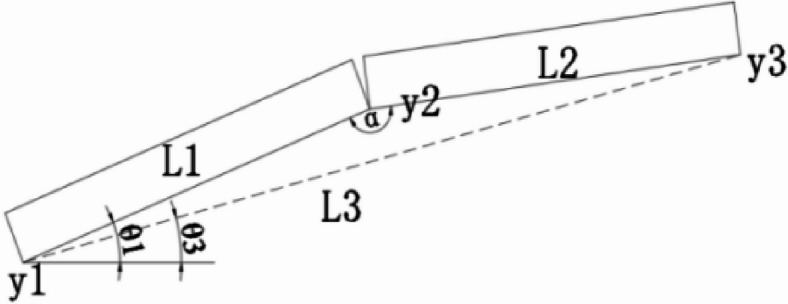
Manufactured line type.

**Fig. 6:**
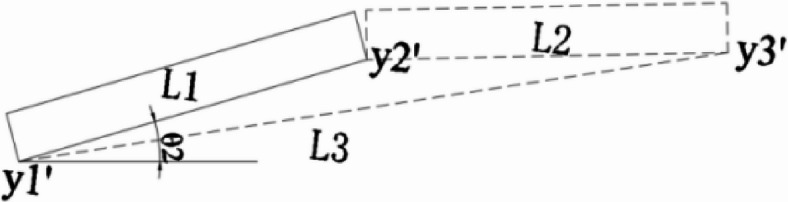
Assembled line type.

Prior to precise alignment, the already installed segments are measured. Based on the relative position of the manufacturing shape, the accurate position of the segment to be installed is calculated. Figure 5 shows the theoretical position of the steel beam segment considering pre-camber, Fig. 6 shows the actual position of the steel beam segment during construction, L1 represents the installed segment, and L2 represents the beam segment to be installed.

The control elevation for the on-site assembly segments can be derived from the manufacturing shape.

$$\normalsize {\theta _1}{\text{=}}arcsin \frac{{{y_2} - {y_1}}}{{{L_1}}}$$ $$\normalsize {\theta _2}{\text{=}}arcsin \frac{{{{y^{\prime}}_2} - {{y^{\prime}}_1}}}{{{L_1}}}$$ $$\normalsize {\theta _3}{\text{=}}arcsin \frac{{{y_3} - {y_1}}}{{{L_3}}}$$

$${y^{\prime}_3}={y^{\prime}_1}+{L_3}\sin ({\theta _3}+{\theta _2} - {\theta _1})$$

$${L_3}=\sqrt {L_{1}^{2}+L_{2}^{2} - 2{L_1}{L_2}\cos \alpha }$$

Since$$\alpha\rightarrow180^{\circ},\cos\alpha\rightarrow-1$$, therefore$${L_3} \approx {L_1}+{L_2}$$

1 $$y^{\prime}_3=y^{\prime}_1+(L_1+L_2)\sin(\theta_3+\theta_2-\theta_1)$$

Where:

${L_1}$—Length of installed segments

${L_2}$—Length of segment to be installed

${y_1}$、${y_2}$、${y_3}$—The theoretical elevation considering manufacturing pre-camber

${y^{\prime}_1}$、${y^{\prime}_2}$、${y^{\prime}_3}$—Actual installation elevation

Based on the above analysis, the theoretical spatial relative position of the steel girder is fixed. Although the measured elevation of the steel girder continuously changes due to environmental influences, as long as the immediate measurement data is used for calculation at the moment of precise alignment, and the assembly elevation is calculated, adjusted, and locked in real time, followed by timely re-measurement and correction, the goal of achieving precise alignment construction of the steel box girder at any time can be realized.

### Error calculation

During construction, errors are inevitable, and thus, error correction is necessary during the precision alignment installation. The error factors that need to be considered mainly include process errors, tangent assembly errors, and measurement timing errors, as well as the influence of cross-sectional deformation.

#### Process errors

For full section welded steel structures, the width of the weld seam is generally the same, and the shrinkage of the weld seam is also basically the same. For composite beam structures, the upper part is a concrete beam and the lower part is a steel beam. When welding the lower steel beam, there will be some shrinkage, while the connection part of the upper concrete structure is cast-in-place, which causes the steel beam to rotate downward. This error should be obtained through experimental measurement.

By analyzing the measured data from the already assembled beam segments, the error caused by the influence of the construction process can be calculated.

After the precision alignment is completed and before welding, the elevation of the beam segment before welding is${y_{21}}$、${y_{22}}$、${y_{23}}$, and the elevation of the beam segment after welding is${y^{\prime}_{21}}$、${y^{\prime}_{22}}$、${y^{\prime}_{23}}$. In theory, the spatial relationship between the three should be consistent. However, due to errors caused by welding deformation and other construction process reasons, the spatial posture changes. The calculation formula for the process error is:

2 $$\Delta y={y^{\prime}_{23}} - \left[ {{{y^{\prime}}_{21}}+{L_3}\sin ({\theta _3}+{\theta _2} - {\theta _1})} \right]$$

Where: $${\theta _1}{\text{=}}arcsin \frac{{{y_{22}} - {y_{21}}}}{{{L_1}}}$$ $${\theta _2}{\text{=}}arcsin \frac{{{{y^{\prime}}_{22}} - {{y^{\prime}}_{21}}}}{{{L_1}}}$$ $${\theta _3}{\text{=}}arcsin \frac{{{y_{23}} - {y_{21}}}}{{{L_3}}}$$

#### Tangent assembly errors

**Fig. 7:**
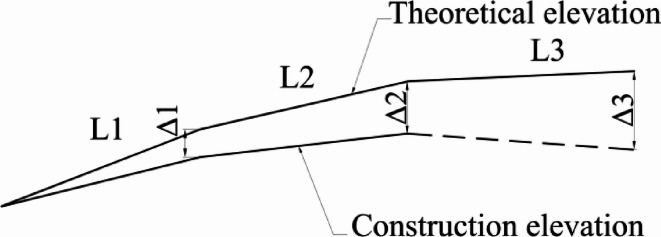
Tangent installation error.

The currently installed beam segments have already developed vertical errors. Continuing to assemble the subsequent beam segments according to the manufacturing shape will lead to an amplification of these errors, so correction must be made during the positioning of the beam segments. (Fig. 7)

Based on the measurement data, the errors of the installed beam segments are calculated to be △1 and △2, and the lengths of the beam segments are L1 and L2、L3. The error of the beam segment to be installed is:

3 $$\Delta 3{\text{=}}\Delta 1+\frac{{L2+L3}}{{L2}}(\Delta 2 - \Delta 1)$$

It is necessary to perform error correction on the elevation of the L3 beam segment to ensure that the line type error does not infinitely amplify.

#### Measurement timing errors

**Fig. 8:**
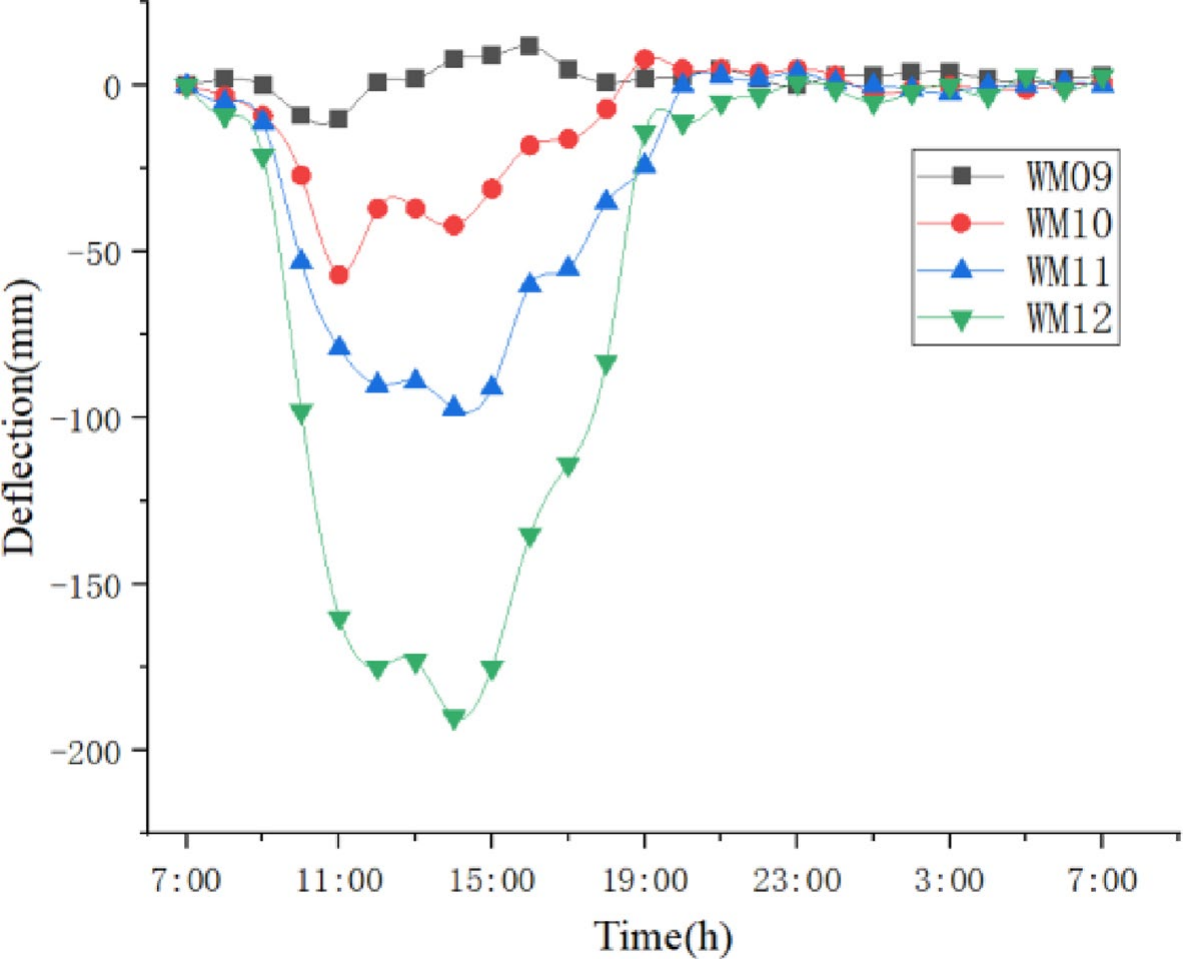
Elevation variation curve of adjacent segments.

**Fig. 9:**
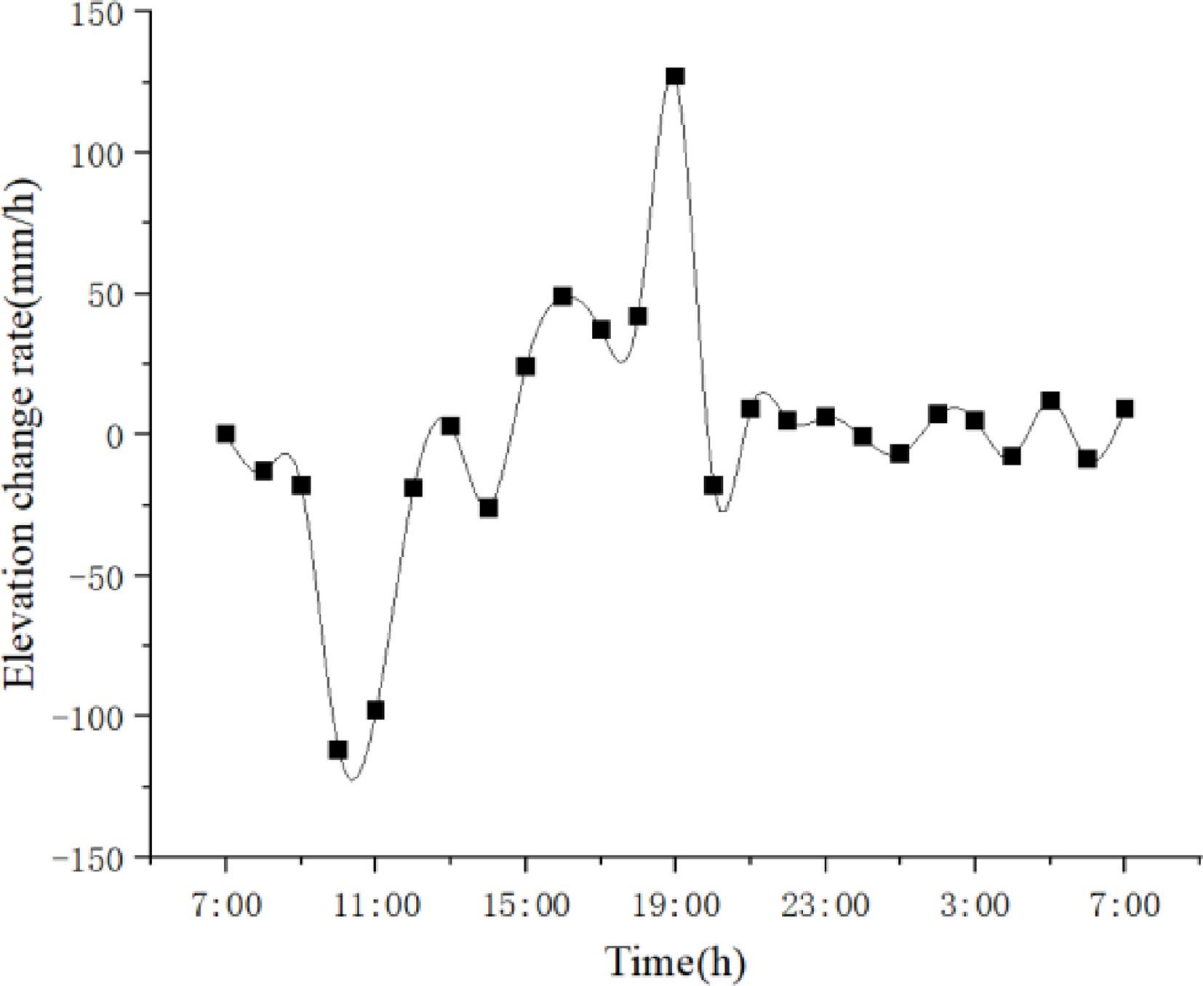
Elevation change rate of the beam segment to be installed.

We use Topcon DL-501 digital level to measure elevation, with a measurement accuracy of 0.2 mm. Nonetheless, due to the time interval between the elevation measurement and the precision alignment of the elevation, the elevation of the beam segments may also change during this interval. This error value is calculated based on the dynamic variation pattern of the elevations of adjacent beam segments. Analysis of the measurement data shows (Figs. 8 and 9) that between 9:00 and 11:00, and 17:00 and 19:00, the elevation change rate of the beam to be assembled is relatively high, while the change rate during other time periods is lower. Therefore, the precision alignment should avoid these time periods. Additionally, this data is significantly affected by weather conditions, and different measurement data should be selected for calculations based on varying weather conditions.

Measurement timing errors can be calculated by formula (4):

4 $$\Delta {H_t}{\text{=}}\Delta {h_t}T$$

Where:

- $\Delta {h_t}$- rate of elevation change of the beam segment to be assembled;
- *T*- time interval.

#### Cross-sectional deformation

**Fig. 10:**
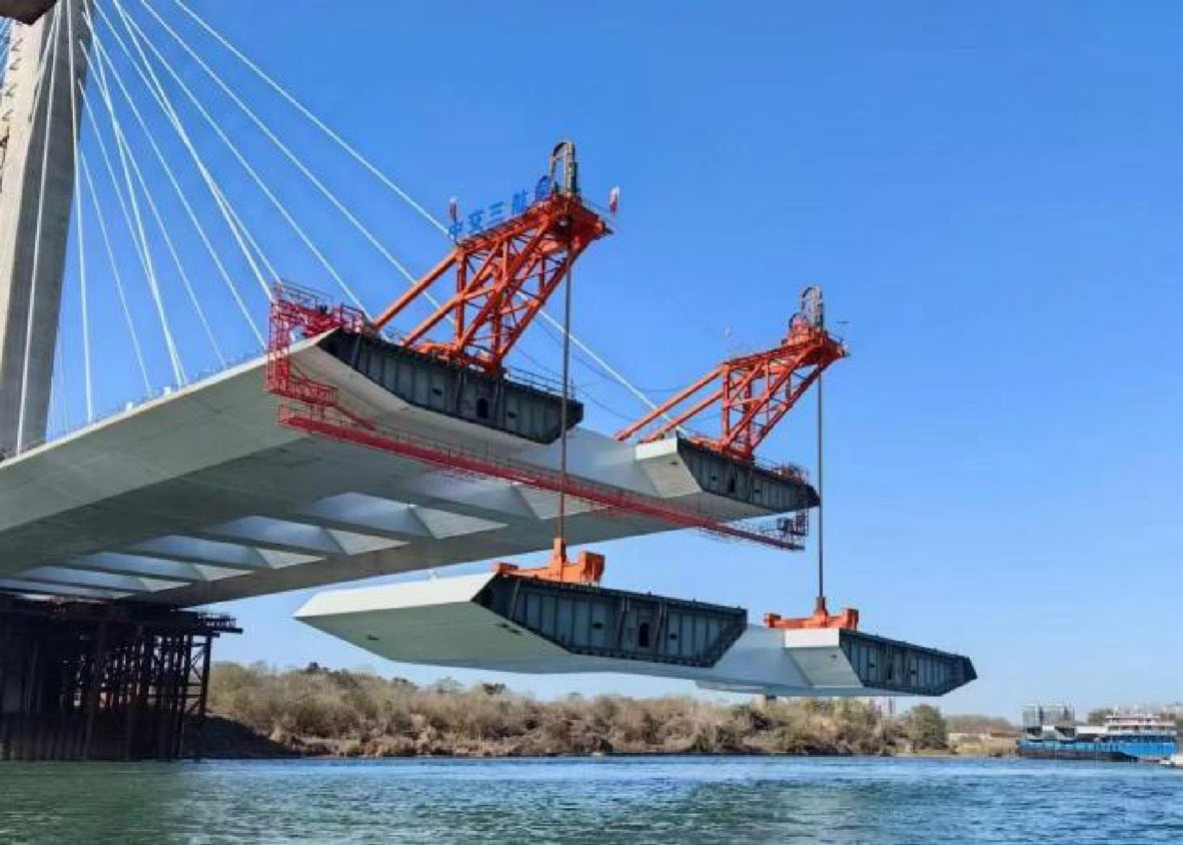
Lifting of steel beam segments.

The standard cross-section of the steel box girder has a 2% cross slope on both sides and is installed using a bridge deck crane (Fig. 10).

To simulate the lateral deformation of steel beam segments, a finite element model was established using Midas FEA NX (Version 2022R1, available at: https://product.midasit.cn/index/). The material used in the model is Q345, vertical constraints were set at the anchor points of the stay cables and the vertical suspension points. The maximum stress of the steel beam to be installed was 108.4 MPa, and the maximum stress of the installed beam was 180.8 MPa, both of which were less than the steel stress design value of 275 MPa.

**Fig. 11:**
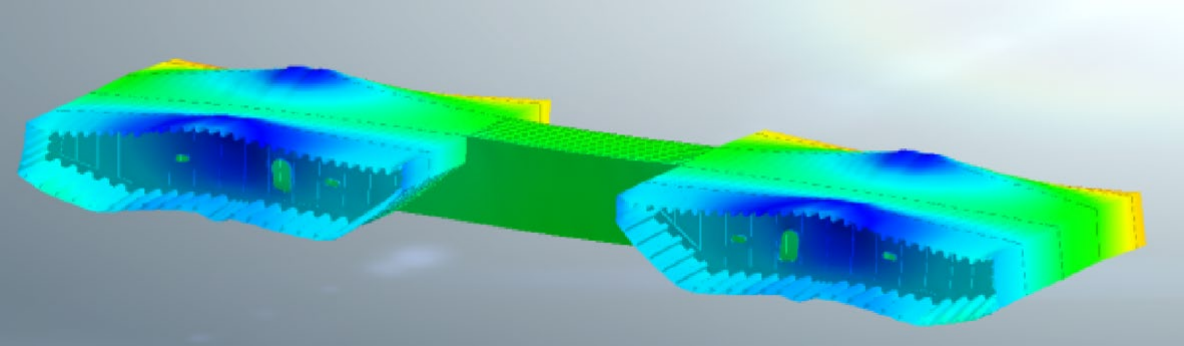
Deformation of segment to be installed.

**Fig. 12:**
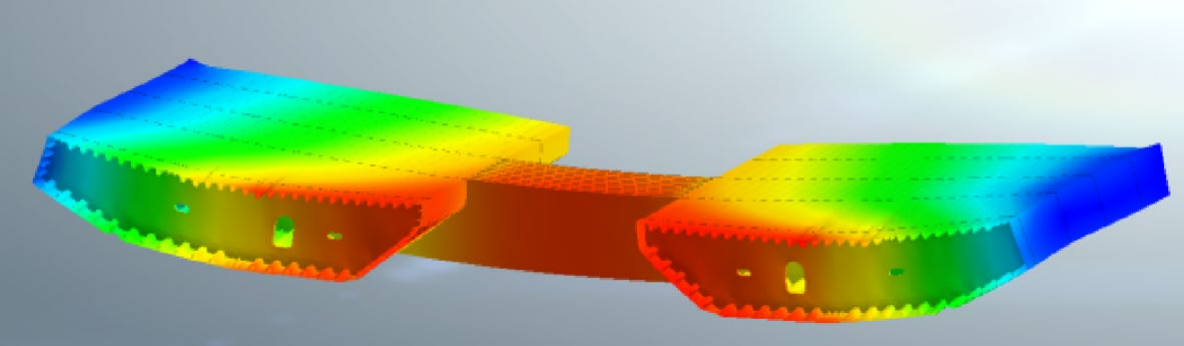
Deformation of installed segment.

**Fig. 13:**
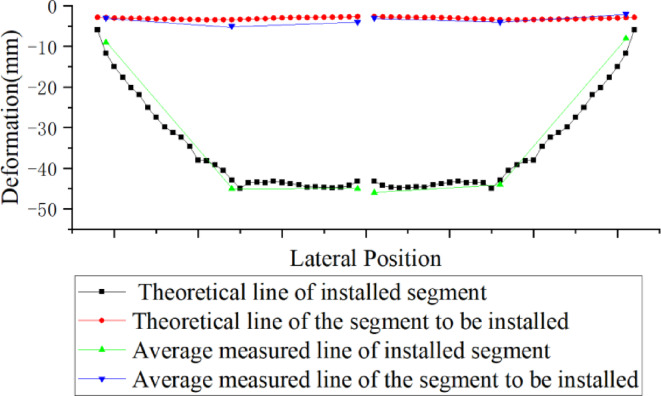
Transverse deformation curve of steel beam.

After the installation of the steel beams, lateral deformation will occur due to the loads from the cables on both sides and the crane loads^20–23^ (Figs. 11 and 12). Therefore, when evaluating the structural alignment, the impact of lateral deformation should be considered. For steel beams lifted by the crane, the elevation of the lifting points may be slightly higher (Fig. 13). The stay cables are located at the outer web of the steel beam, while the crane is positioned at the midsection of the steel box beam. Once the stay cables are tensioned, upward warping deformation will occur on both sides of the steel box beam. The lateral deformation of the cross-section should be calculated before construction, and actual measurements should be taken during the construction process to allow for elevation adjustments. Since the outer web is the primary load-bearing component, when matching the beam segments, the elevation of the outer web is matched first, followed by the adjustment of the relative height differences at other positions. Alternatively, the cross-sectional matching height difference can be reduced by pre-tensioning the stay cables to transfer the lifting weight^24^.

## Result

**Fig. 14:**
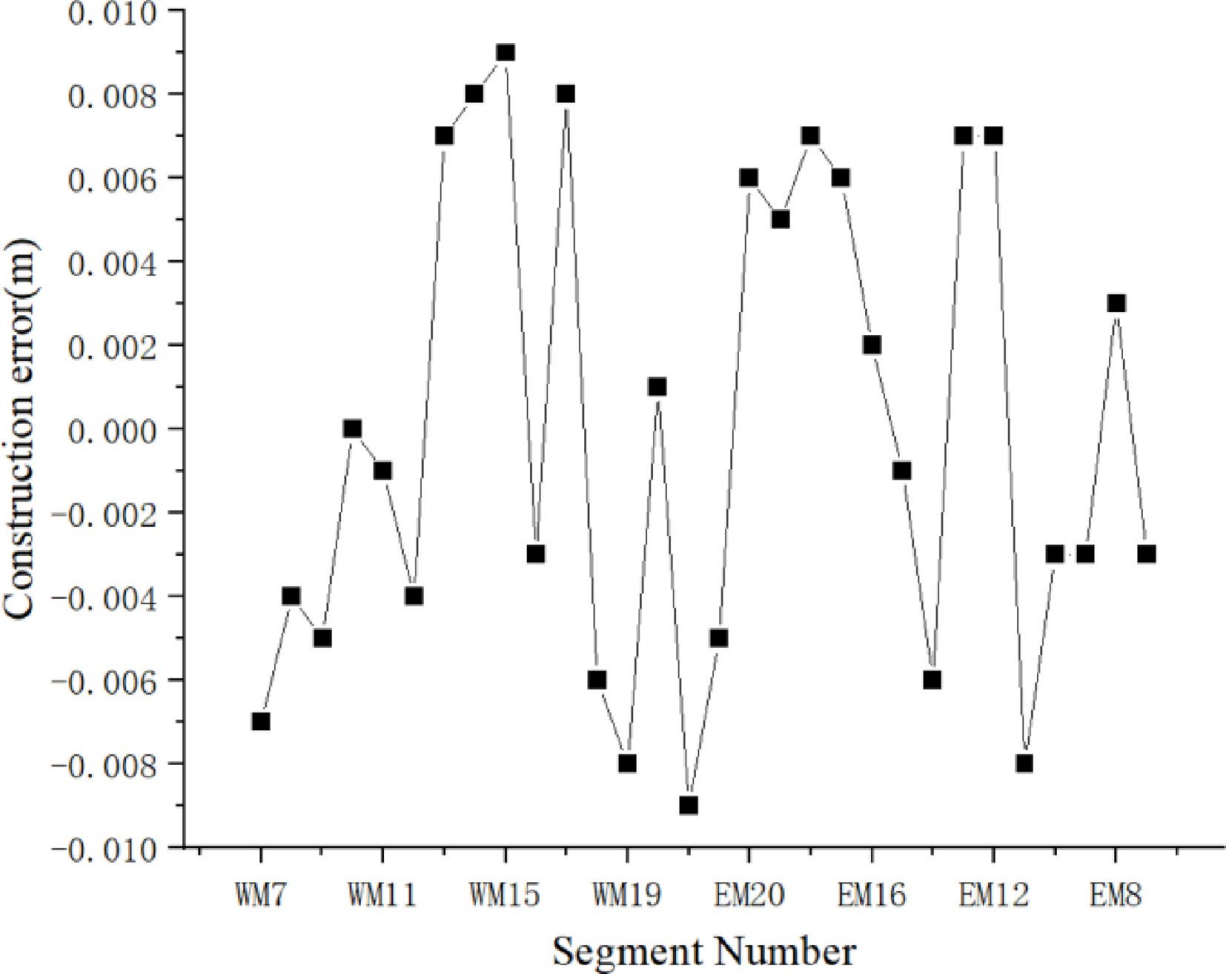
Result of dynamic precise alignment.

According to the method described in this article, daytime dynamic precision alignment was performed on the WM7-WM21 and EM7-EM21 beam segments, covering a time span from November 2023 to May 2024, experiencing extreme weather conditions in winter and summer, with temperature changes ranging from − 5 ℃ to 30 ℃. The precision alignment error value calculated from the elevation measurement data under stable temperature conditions before the installation of the stay cables after the completion of the steel beam welding is shown in Fig. 14. As shown in the data in the figure, including welding deformation, precision adjustment errors and process errors, the dynamic precision matching error range of the steel beam is between − 9 mm and 9 mm, meeting the requirement of assembly error less than 20 mm in the specification.

## Discussing

**Fig. 15:**
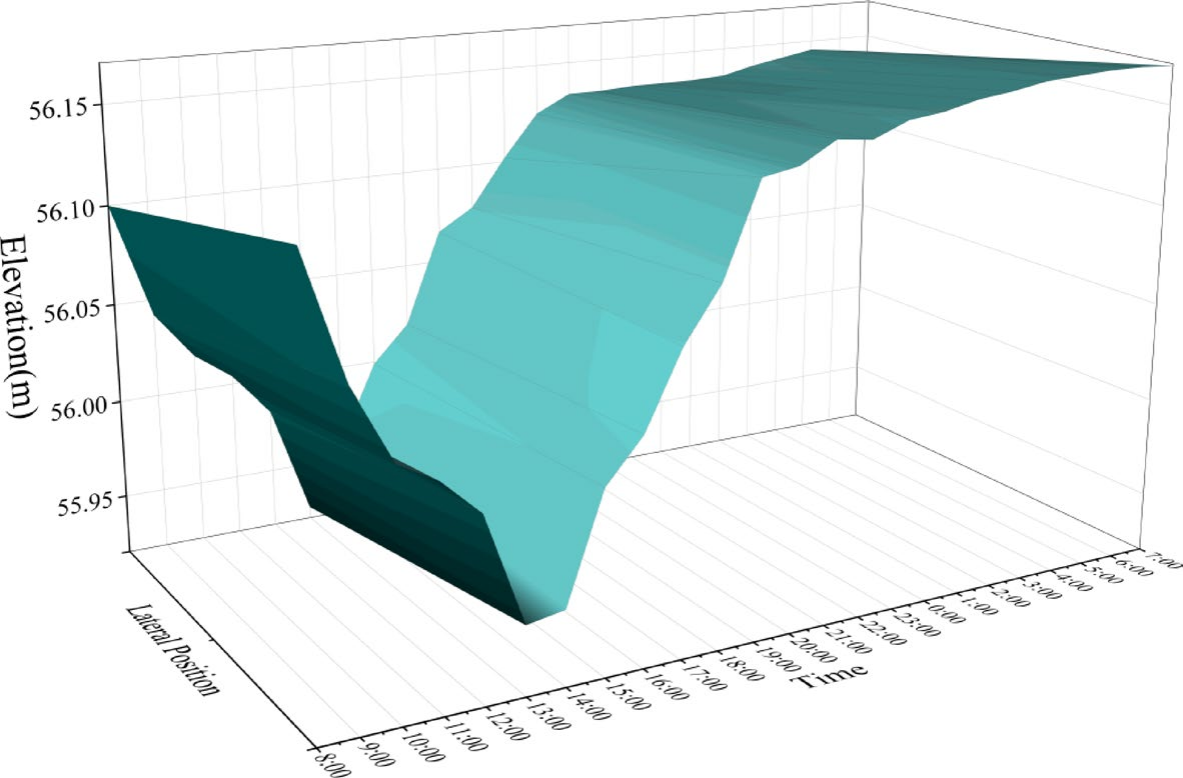
Elevation of segment WM20.

**Fig. 16:**
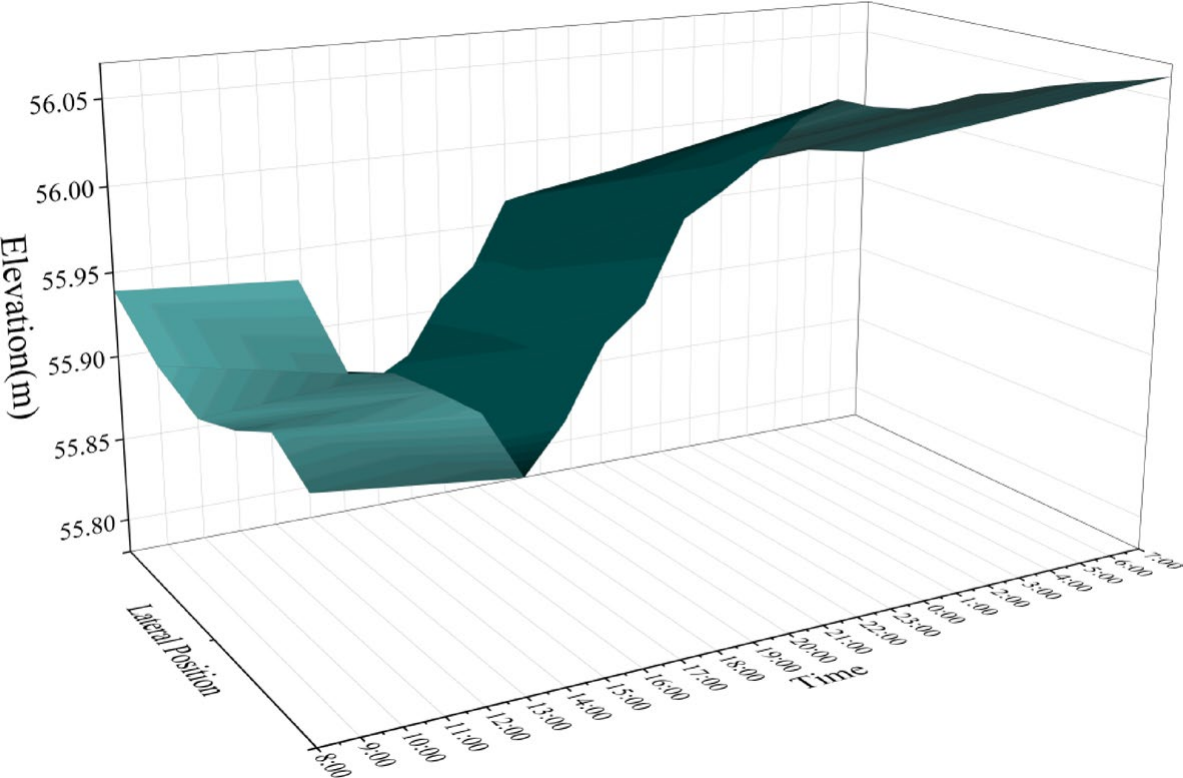
Elevation of segment EM20.

We measured the elevation of WM20 and EM20 beam segments from 7:00 am on May 7, 2024 to 7:00 am on May 8, 2024, and their elevations are shown in Figs. 15 and 16. The “lateral position” axis represents the position of the measuring point in the transverse direction of the bridge, and the “time” axis represents the measurement time with a time interval of 1 h. it can be found that:

(1) The elevation of the main beam is affected by environmental factors such as sunlight and temperature, and varies greatly between day and night, with a maximum variation of 21.9 cm. The elevation reaches its lowest value at 13:00 during the day and its maximum value at 5:00 in the morning.

(2) During the period of 7:00–19:00, the elevation changes dramatically, with an average rate of change reaching 36.5 mm/h. The elevation changes relatively smoothly from 00:00 to 7:00, with an average rate of change of 3.3 mm/h.

(3) The elevation of the left and right sides is affected by uneven sunlight, and there are differences in elevation. The maximum deviation between the left and right sides reaches 37 mm.

**Fig. 17:**
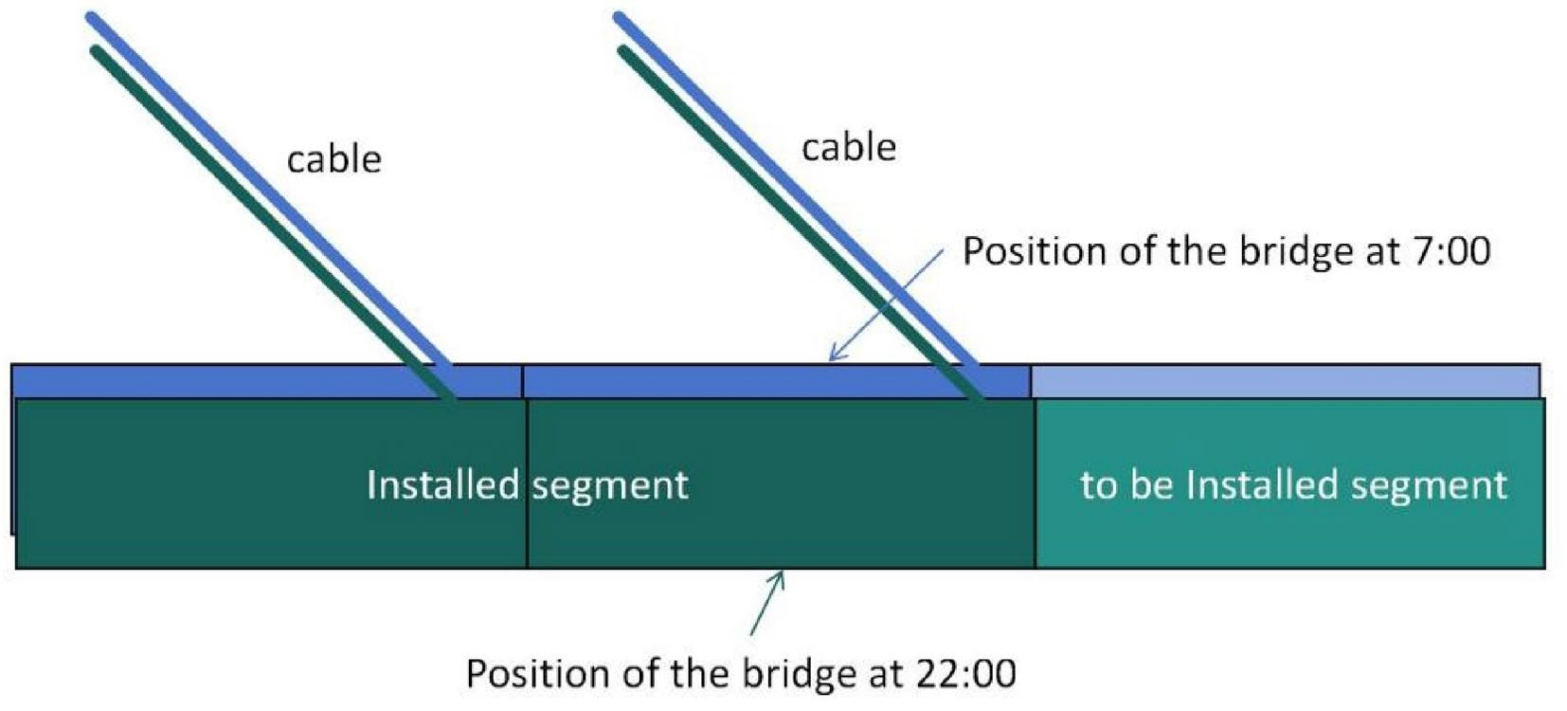
Static precise matching elevation at different times at night.

The precise alignment and positioning of the WM20 beam segment were carried out under this temperature condition. According to the calculation results, the precise alignment construction was carried out based on the measurement data at 22:00. By 7:00 the next morning, According to the algorithm above, the actual theoretical precise alignment elevation had changed from 55.961 m to 55.986 m, with a variation amplitude of 25 mm (Fig. 17). Therefore, under the condition of drastic changes in elevation, traditional precise alignment cannot meet the requirements of construction control. Even during periods of relatively stable nighttime temperatures, the precise alignment elevation will undergo significant changes.

## Conclusion

The precision requirements for the precise alignment construction of steel box girders are high. By establishing a rapid precise alignment positioning algorithm, three measurements are taken to calculate the respective precise positioning elevations, which are used to position and adjust the posture of the steel box girder. The first measurement is used for welding the web cleats, achieving initial fixation; the second measurement is used to verify the relative positional accuracy after the web cleats have been welded and to further adjust the elevation. The third measurement is used to assess the process error, serving as the basis for precise alignment calculations. Following the method described in this paper for the precise alignment construction of steel box girders, the precision can meet the requirements of construction specifications.

(1) The method presented in this paper breaks through the traditional regulation that precise alignment construction can only be measured and carried out at night, allowing for the precise alignment construction of steel girders to be conducted during the day. This avoids the risks associated with nighttime construction safety and welding quality, and can also accelerate the construction schedule to some extent.

(2) Nighttime temperatures are not absolutely stable, and the elevation of steel girders can also undergo minor changes. Using traditional precise alignment methods, the influence of this error still cannot be excluded. The method in this paper can comprehensively consider the dynamic deformation of the bridge and process errors at any time, thereby circumventing the impact of this error and improving the control precision of precise alignment.

(3) Due to the fact that the method proposed in this article is based on rapid measurement and calculation, there is a high demand for efficiency in measurement and calculation during construction.

(4)Further research is needed on how to quickly adjust the elevation of steel girders during precise matching of steel box girders and reduce the changes in steel girders elevation during the adjustment period.

## Electronic supplementary material

Below is the link to the electronic supplementary material.

Supplementary Material 1

## Data Availability

The datasets generated and analyzed during the current study are available from the corresponding author on reasonable request. Data supporting the findings of this study are also included within supplementary materials.
