# Supplementary material for: All-weather precision alignment technology for ultra-wide steel box girders in cable-stayed bridges
Source: Sci Rep. 2025 Jul 11;15:25062. doi: 10.1038/s41598-025-08562-6 (PMC12254333; doi:10.1038/s41598-025-08562-6)
Supplement: Supplementary file 1 — Supplementary Material 1 [file 41598_2025_8562_MOESM1_ESM.docx]

**Raw date of this manuscript**

# （1）Fig.6 Elevation variation curve of adjacent segments

mm

| Time | WM09 | WM10 | WM11 | WM12 |
| --- | --- | --- | --- | --- |
| 7:00:00 | 0 | 0 | 0 | 0 |
| 8:00:00 | 2 | -3 | -5 | -9 |
| 9:00:00 | 0 | -9 | -11 | -21 |
| 10:00:00 | -9 | -27 | -53 | -98 |
| 11:00:00 | -10 | -57 | -79 | -160 |
| 12:00:00 | 1 | -37 | -90 | -175 |
| 13:00:00 | 2 | -37 | -89 | -173 |
| 14:00:00 | 8 | -42 | -97 | -190 |
| 15:00:00 | 9 | -31 | -91 | -175 |
| 16:00:00 | 12 | -18 | -60 | -135 |
| 17:00:00 | 5 | -16 | -55 | -114 |
| 18:00:00 | 1 | -7 | -35 | -83 |
| 19:00:00 | 2 | 8 | -24 | -14 |
| 20:00:00 | 3 | 5 | 0 | -11 |
| 21:00:00 | 5 | 5 | 3 | -5 |
| 22:00:00 | 2 | 4 | 2 | -3 |
| 23:00:00 | 0 | 5 | 4 | 1 |
| 0:00:00 | 3 | 3 | 1 | -1 |
| 1:00:00 | 3 | -2 | 0 | -5 |
| 2:00:00 | 4 | -1 | -1 | -2 |
| 3:00:00 | 4 | 0 | -2 | 0 |
| 4:00:00 | 2 | -1 | 0 | -3 |
| 5:00:00 | 1 | -1 | 0 | 3 |
| 6:00:00 | 2 | 0 | 1 | -1 |
| 7:00:00 | 3 | 1 | 0 | 3 |


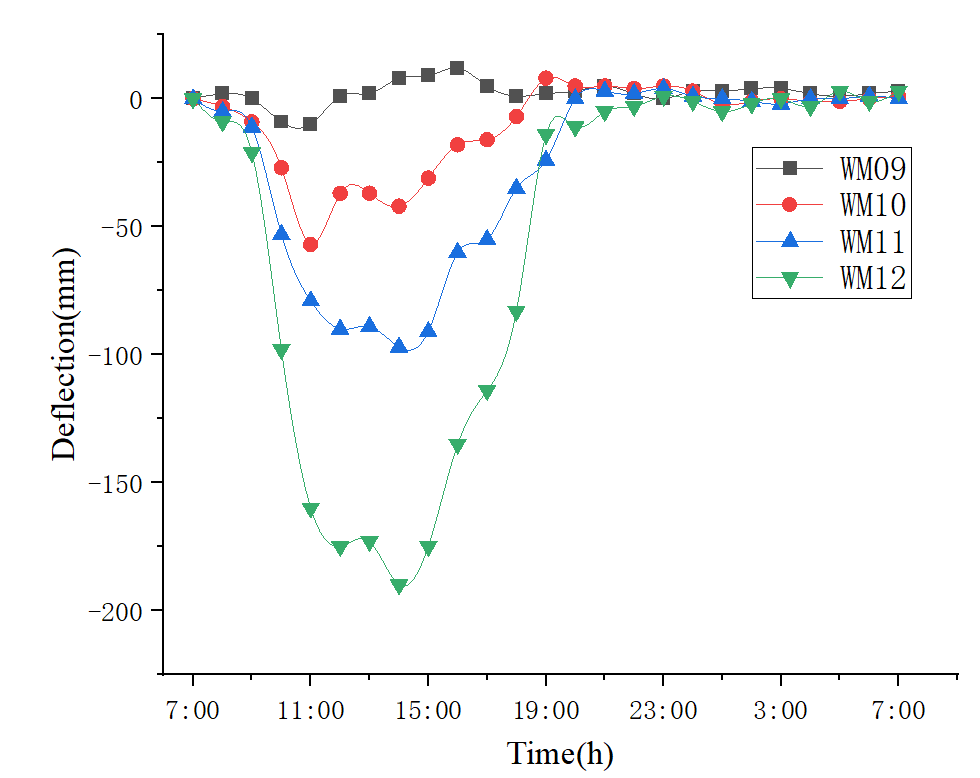


Fig.6 Elevation variation curve of adjacent segments

# （2）Fig.7 Elevation change rate of the beam segment to be installed

mm/h

| Time | rate | Time | rate |
| --- | --- | --- | --- |
| 7:00 | 0 | 20:00 | -18 |
| 8:00 | -13 | 21:00 | 9 |
| 9:00 | -18 | 22:00 | 5 |
| 10:00 | -112 | 23:00 | 6 |
| 11:00 | -98 | 0:00 | -1 |
| 12:00 | -19 | 1:00 | -7 |
| 13:00 | 3 | 2:00 | 7 |
| 14:00 | -26 | 3:00 | 5 |
| 15:00 | 24 | 4:00 | -8 |
| 16:00 | 49 | 5:00 | 12 |
| 17:00 | 37 | 6:00 | -9 |
| 18:00 | 42 | 7:00 | 9 |
| 19:00 | 127 |  |  |


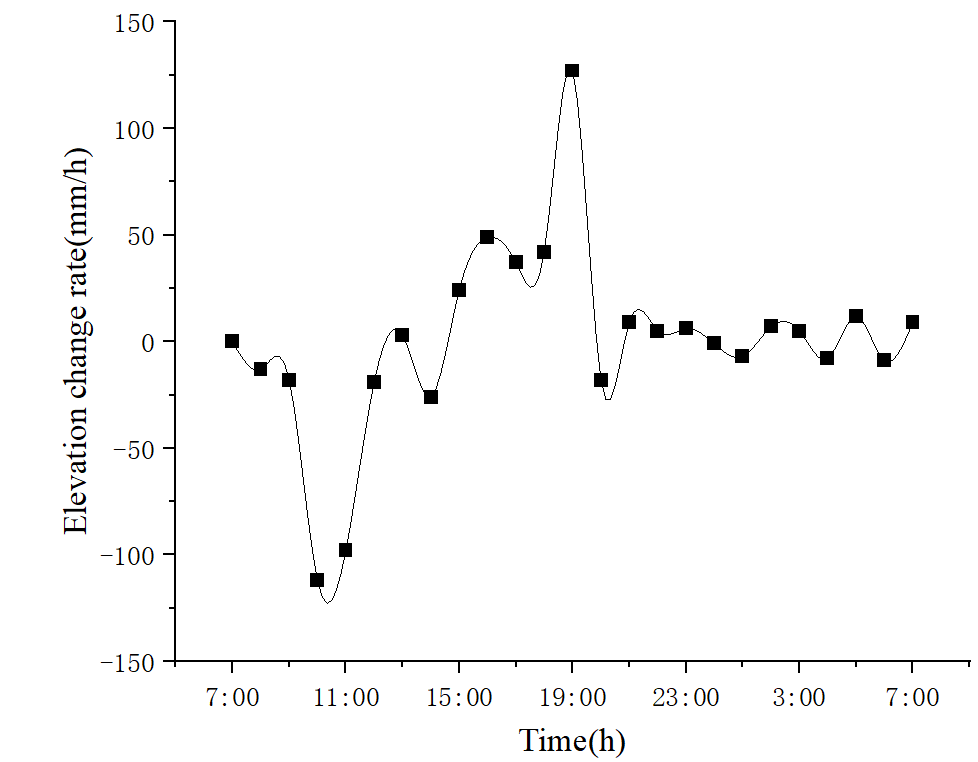


Fig.7 Elevation change rate of the beam segment to be installed

# （3）Fig.11 Transverse deformation curve of steel beam

mm

| Lateral Position | Installed segment | Segment to be installed |
| --- | --- | --- |
| 12 | -43.5071 | -3.1411 |
| 13 | -43.3831 | -3.2032 |
| 14 | -43.5562 | -3.272 |
| 15 | -44.9324 | -3.3352 |
| 16 | -42.8783 | -3.4036 |
| 17 | -40.581 | -3.431 |
| 18 | -39.1512 | -3.4405 |
| 19 | -38.1107 | -3.4275 |
| 20 | -38.0516 | -3.3777 |
| 21 | -34.5519 | -3.3386 |
| 22 | -32.3129 | -3.3106 |
| 23 | -31.1888 | -3.2749 |
| 24 | -29.8167 | -3.2447 |
| 25 | -27.4697 | -3.2164 |
| 26 | -25.0914 | -3.1818 |
| 27 | -21.9466 | -3.0536 |
| 28 | -20.1585 | -3.1042 |
| 29 | -17.5982 | -3.0612 |
| 30 | -14.964 | -3.0145 |
| 31 | -11.6867 | -2.9077 |
| 32 | -5.8843 | -2.8163 |


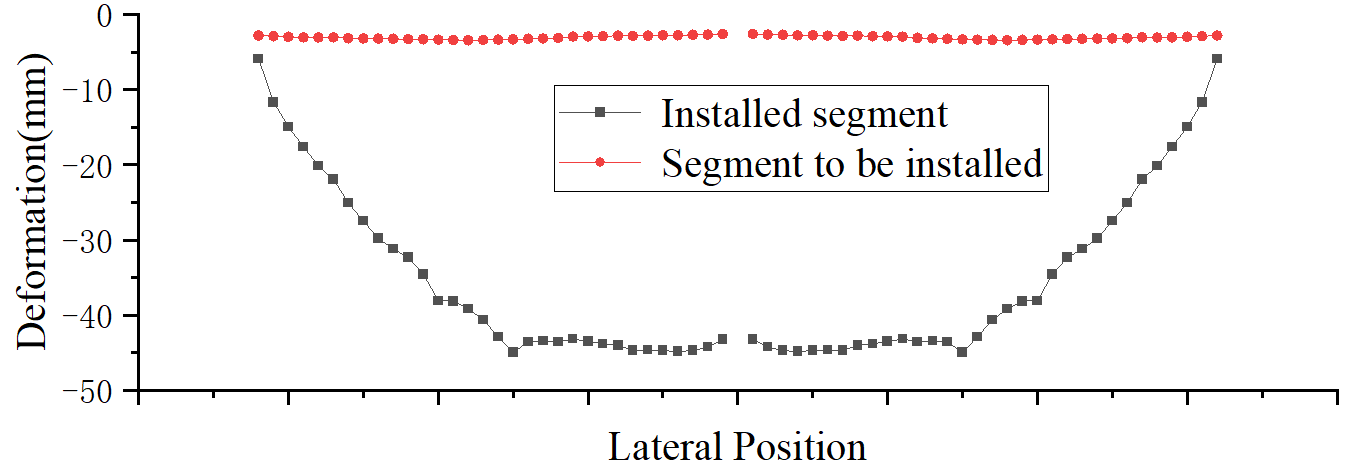


Fig.11 Transverse deformation curve of steel beam

# （4）Fig.12 Result of dynamic precise alignment

m

| Segment Number | Error according to the method described in this article | Segment Number | Error according to the method described in this article |
| --- | --- | --- | --- |
| WM7 | -0.007 | EM21 | -0.005 |
| WM8 | -0.004 | EM20 | 0.006 |
| WM9 | -0.005 | EM19 | 0.005 |
| WM10 | 0 | EM18 | 0.007 |
| WM11 | -0.001 | EM17 | 0.006 |
| WM12 | -0.004 | EM16 | 0.002 |
| WM13 | 0.007 | EM15 | -0.001 |
| WM14 | 0.008 | EM14 | -0.006 |
| WM15 | 0.009 | EM13 | 0.007 |
| WM16 | -0.003 | EM12 | 0.007 |
| WM17 | 0.008 | EM11 | -0.008 |
| WM18 | -0.006 | EM10 | -0.003 |
| WM19 | -0.008 | EM9 | -0.003 |
| WM20 | 0.001 | EM8 | 0.003 |
| WM21 | -0.009 | EM7 | -0.003 |


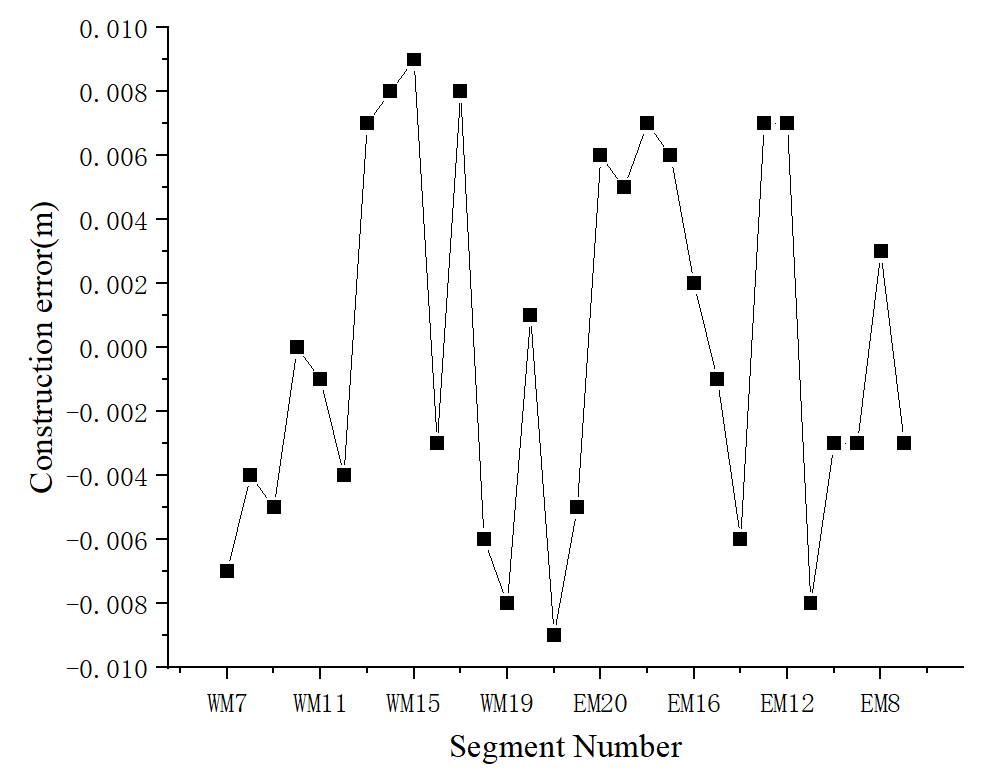


Fig.12 Result of dynamic precise alignment

# Fig.13 Elevation of segment L21

m

| Time | Left side | Right side |
| --- | --- | --- |
| 7:00 | 56.142 | 56.168 |
| 8:00 | 56.1 | 56.135 |
| 9:00 | 56.042 | 56.075 |
| 10:00 | 56.018 | 56.038 |
| 11:00 | 56.004 | 56.027 |
| 12:00 | 55.982 | 56.009 |
| 13:00 | 55.926 | 55.954 |
| 14:00 | 55.959 | 55.957 |
| 15:00 | 55.999 | 56.01 |
| 16:00 | 56.018 | 56.031 |
| 17:00 | 56.066 | 56.068 |
| 18:00 | 56.077 | 56.094 |
| 19:00 | 56.102 | 56.139 |
| 20:00 | 56.124 | 56.142 |
| 21:00 | 56.133 | 56.152 |
| 22:00 | 56.133 | 56.15 |
| 23:00 | 56.134 | 56.157 |
| 0:00 | 56.134 | 56.159 |
| 1:00 | 56.134 | 56.163 |
| 2:00 | 56.135 | 56.165 |
| 3:00 | 56.139 | 56.168 |
| 4:00 | 56.142 | 56.169 |
| 5:00 | 56.145 | 56.17 |
| 6:00 | 56.143 | 56.17 |
| 7:00 | 56.141 | 56.169 |


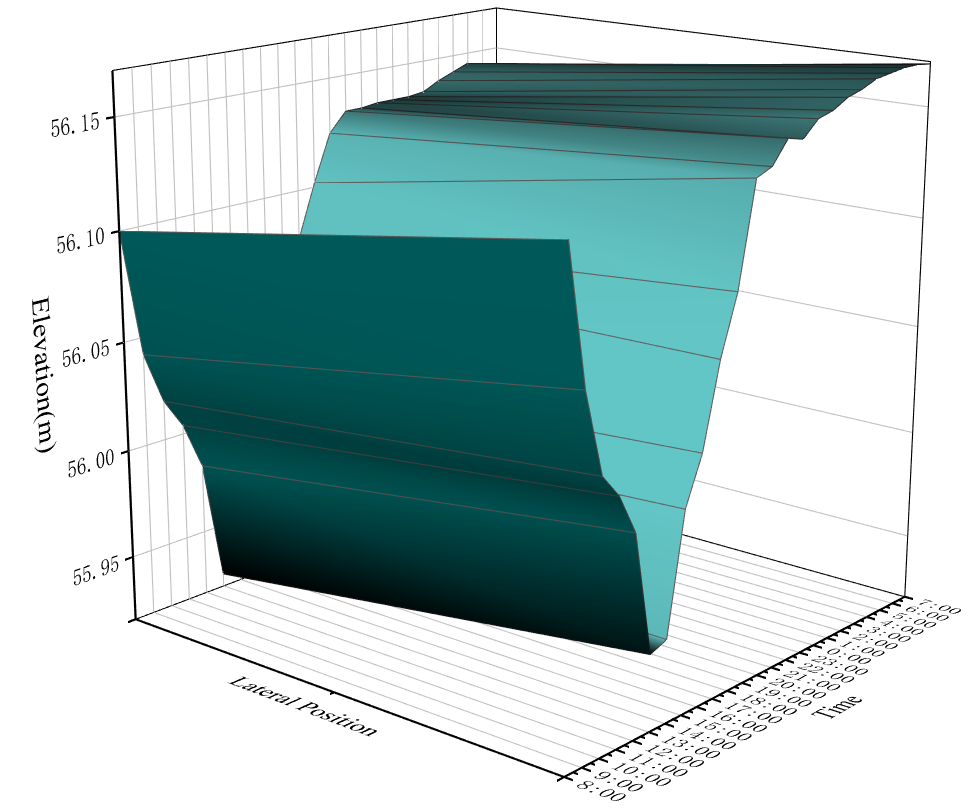


Fig.13 Elevation of segment L21

# （6）Fig.14 Elevation of segment L25

| Time | Left side | Right side |
| --- | --- | --- |
| 7:00 | 56.059 | 55.969 |
| 8:00 | 56.013 | 55.939 |
| 9:00 | 55.965 | 55.891 |
| 10:00 | 55.962 | 55.855 |
| 11:00 | 55.949 | 55.844 |
| 12:00 | 55.935 | 55.839 |
| 13:00 | 55.898 | 55.796 |
| 14:00 | 55.923 | 55.805 |
| 15:00 | 55.96 | 55.862 |
| 16:00 | 55.977 | 55.874 |
| 17:00 | 56.018 | 55.907 |
| 18:00 | 56.03 | 55.925 |
| 19:00 | 56.044 | 55.964 |
| 20:00 | 56.062 | 55.968 |
| 21:00 | 56.07 | 55.97 |
| 22:00 | 56.064 | 55.966 |
| 23:00 | 56.061 | 55.973 |
| 0:00 | 56.062 | 55.973 |
| 1:00 | 56.065 | 55.972 |
| 2:00 | 56.064 | 55.971 |
| 3:00 | 56.065 | 55.97 |
| 4:00 | 56.065 | 55.972 |
| 5:00 | 56.064 | 55.974 |
| 6:00 | 56.063 | 55.971 |
| 7:00 | 56.063 | 55.968 |


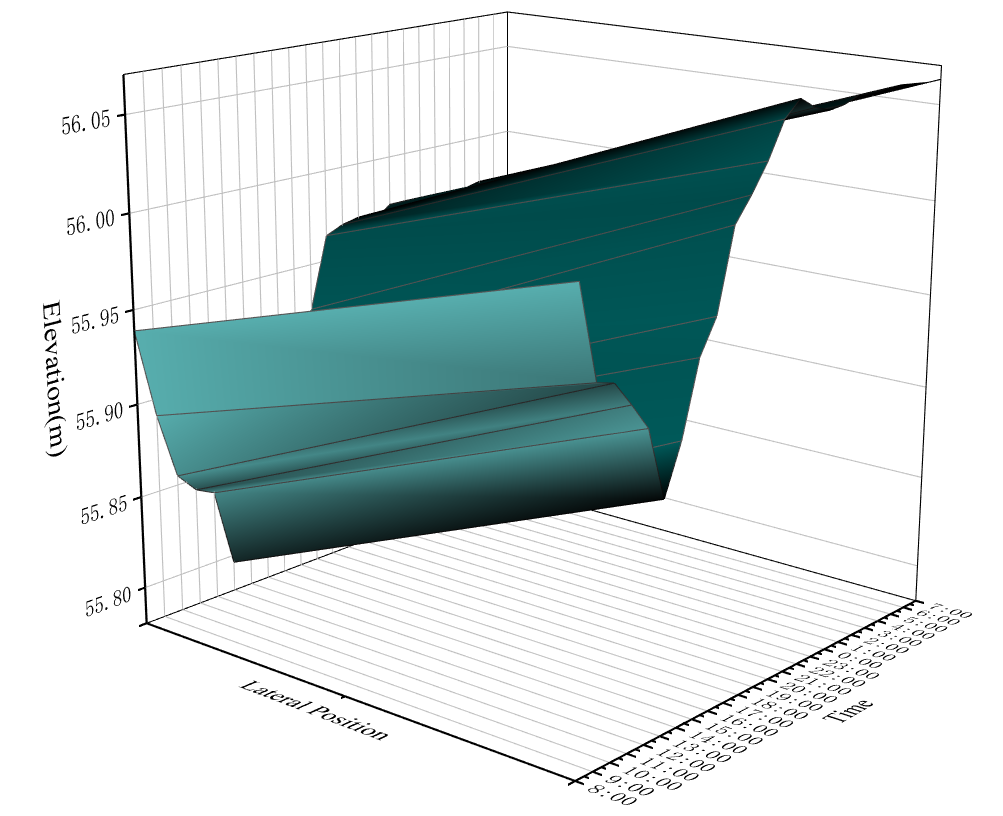


Fig.14 Elevation of segment L25

# Fig.15 Static precise matching elevation at different times at night

m

| time | 22:00 | 7:00 |
| --- | --- | --- |
| L19 | 56.4555 | 56.465 |
| L20 | 56.2775 | 56.2895 |
| L21 | 56.1415 | 56.155 |
| L22 | 55.961 | 55.986 |


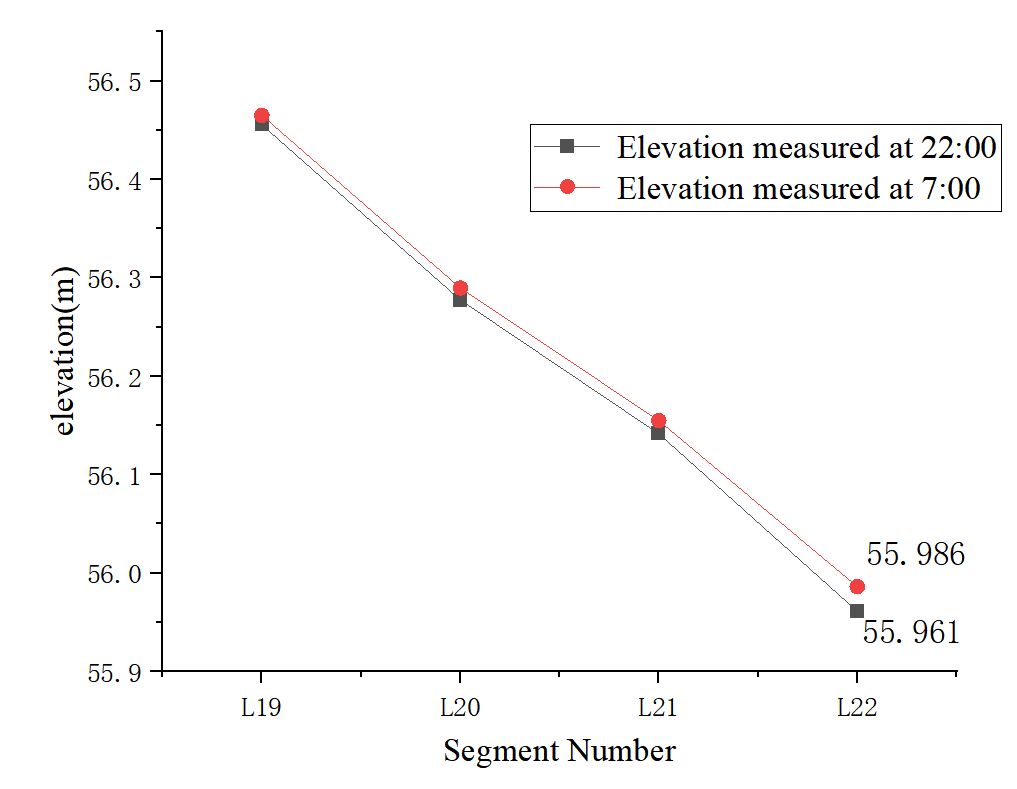


Fig.15 Static precise matching elevation at different times at night
